# Evaluating a Memory Clinic Using the RE-AIM Model. The Experience of the “Memory and Neuropsychiatry Clinic” in Hospital Del Salvador, Chile

**DOI:** 10.3389/fneur.2021.612416

**Published:** 2021-09-06

**Authors:** Tomas Leon, Loreto Castro, Franco Mascayano, Brian Lawlor, Andrea Slachevsky

**Affiliations:** ^1^Memory and Neuropsychiatric Clinic, Neurology Department, Del Salvador Hospital and University of Chile School of Medicine, Santiago, Chile; ^2^Department of Psychiatry and Global Brain Health Institute, Trinity College, Dublin, Ireland; ^3^Mailman School of Public Health, Columbia University, New York, NY, United States; ^4^Department of Psychiatry, New York State Psychiatric Institute, New York, NY, United States; ^5^Geroscience Center for Brain Health and Metabolism (GERO), Santiago, Chile; ^6^Neuropsychology and Clinical Neuroscience Laboratory (LANNEC), Physiopathology Department, Instituto de Ciencias Biomedicas (ICBM), Neurosciences and East Campus Neuroscience Departments, University of Chile School of Medicine, Santiago, Chile; ^7^Neurology Unit, Department of Medicine, Clinica Alemana, Universidad del Desarrollo, Santiago, Chile

**Keywords:** memory clinic, public health, Latin America, RE-AIM, implementation

## Abstract

The development of healthcare services for dementia is key to improving access to care and post-diagnostic support for people living with dementia. Memory Units have emerged as a new healthcare service composed of multidisciplinary teams with the goal of improving diagnosis and/or management of dementia patients. The main objective of this study was to describe and evaluate the Reach and Effectiveness of a Memory Unit in a public hospital in Chile, using the RE-AIM model, a multi-component model that allows for the evaluation of the implementation of ongoing healthcare programs. Regarding “R” (Reach): from March 2018 up to June 2019, a total of 510 patients were referred and assessed. Most patients came from primary care (51.9%) and from outpatient services at the Hospital Salvador (39.2%), particularly from the Neurology (63.3%) and Psychiatry (16.0%) departments. We estimated that our Memory Unit assessed 5.39% of all of the dementia patients living in the area of referral. With respect to “E” (Effectiveness): 419 patients are still being followed up at the Memory Unit. Ninety-one patients (18%) were discharged. Of these, 55 (66%) were referred to primary healthcare, 28 (31%) to other outpatient services, 9 (10%) to a specialized mental healthcare center, and 9 (10%) to a daycare center. Due to the short period of time that the Memory Unit has been operating, no other RE-AIM dimensions could be evaluated at this juncture. To our knowledge, this is the first implementation study of a Memory Unit in Latin America, and the first using the RE-AIM model. Although cultural differences worldwide might play a role in the lack of international guidelines, the publication of the experience of the first year of this unit in Chile could inform new countries about this process. Ongoing challenges include continuing to collect data to complement the RE-AIM evaluation and developing a protocol that can be adopted elsewhere in Chile and Latin America. Further studies are needed to assess the benefits of a Memory Unit in comparison to regular care and to develop a model that assures continuity and coordination of care for people with dementia.

## Introduction

Dementia is a syndrome composed of impairment in one or more cognitive domains (e.g., memory, language, orientation, and decision-making) that affects everyday day functioning and independent living. The main causes of dementia include Alzheimer's disease, vascular dementia, and other neurodegenerative disorders (1). The prevalence of dementia in Chile is 1.06%, which means that over 200,000 individuals are living with dementia in the country. If we consider the impact that dementia has on relatives and friends, one can estimate that over 800,000 people are dealing with the consequences of this condition in Chile (2). In those 60 years old and older, the prevalence was 7.0% (women 7.7%; men 5.9%) and was higher in rural than in urban samples (10.3 vs. 6.3%) (3). There is no information on the possible underdiagnosis of dementia in Chile or in Latin America. A recent PRISMA systematic review on diagnosis of dementia only found one Latin America, with no information on underdiagnosis (4).

Chile's population is aging and consequently there is a projected increase in the incidence and prevalence of dementia. It has been estimated that over half a million people will have dementia by 2050 (5, 6). However, the health system is not prepared to tackle the challenge of increasing numbers of people with dementia, with inadequate numbers of dementia specialists, a lack of primary care training in dementia, and low numbers of daycare centers (7–9). There are few studies on the costs of dementia in Chile; however, it has been reported that families with a person living with dementia spend over 1,400 US dollars per month on care, mainly due to indirect costs. This cost is greater for poorer families (10, 11).

Therapies to cure or modify the course of dementia have been unsuccessful so far. Therefore, the main goals of dementia care are to (1) develop preventive strategies, (2) provide timely diagnosis, and (3) provide care and interventions that improve the quality of life of the person with dementia and their caregivers (12).

Due to the complexity of dementia, a comprehensive and holistic multidisciplinary approach is needed. There is evidence that a collaborative care model with a focus on primary care improves several outcomes. For the person with dementia, a collaborative care model results in fewer ER visits and acute hospitalizations (13), an improvement in neuropsychological symptoms (14), and cognitive symptoms (15), earlier diagnosis (16), and better satisfaction with care (17). For caregivers, such a model can improve depressive symptoms (14), decreased burden (18), and result in higher satisfaction with care (17), shorter waiting times and a reduction in time to diagnosis (19, 20).

In this context, the WHO recommends that countries should develop Dementia Plans as a multidisciplinary and multilevel response to tackle the many challenges posed by dementia. At the moment, 28 countries around the world have implemented Dementia Plans, mostly in high-income countries, with only three in Latin America (Costa Rica, Cuba and Chile) (6).

In 2017, the Ministry of Health launched *The Chilean National Plan of Dementia* (hereafter, “the Plan”) for Chile (21) and began its implementation. The plan proposes a model of coordinated care for people with dementia and their caregivers across a continuum from primary care to specialized Memory Units (MU) (22).

MU, also known as Memory Clinics or memory assessment services, were first established in the USA in the 1970 s to provide diagnostic, treatment, and research in dementia (23). MU consist of multidisciplinary teams, bringing together professionals such as neurologists, geriatricians, psychiatrists, psychologists, neuropsychologists, occupational therapists, speech therapists, and nurses (24). MU have been proposed as a more comprehensive service at no extra cost compared to traditional community mental health teams (25); however, there is still a dearth of data supporting the cost-effectiveness of MU as part of the dementia care system (26). Therefore, more research is needed regarding the analysis of the health economic aspects and the implementation of MU.

MU also have a role in providing education and provide training about dementia to primary care, with the goal of improving diagnostic and treatment capacity in primary care settings (27). There have been several reports on the implementation of MU worldwide (28–30) with positive outcomes for patients and caregivers (31), establishing MU as an acceptable and effective form of dementia care (26, 32–34).

A number of MU have been established in Latin America, in private and/or universities centers, mainly in the capital and/or main cities of Argentina, El Salvador, Brazil, and Peru. However, to our knowledge, there is no MU implemented in a network of care facilities within the public system. Additionally, there is little available information on the setup and implementation approaches for such services that can be used as a basis for the development of new MU (35). Although there have been some efforts to develop quality standards for MU (36, 37), these exist mainly in high-income countries (HIC) and are therefore not necessarily applicable to the Chilean population and other low- and middle-income countries (LMIC).

In the context of the Chilean plan, MU are at the level of specialized care in secondary health facilities, based in the main hospitals across the country, one in Santiago (the nation's capital) with the others located in Osorno and Magallanes, regional capitals. Their purpose is to assess people with dementia and their caregivers whose health needs cannot be managed by primary care teams or in community centers for people with dementia, and/or that require evaluation by a dementia specialist (e.g., young-onset, atypical and complex cases with severe and/or treatment resistant symptoms).

An evaluation of the implementation of MU is crucial to inform the development of the Plan and to guide the creation of new units. Such an evaluation should be guided by models that characterize and help understand the implementation processes. One such model is RE-AIM (Reach, Effectiveness, Adoption, Implementation, and Maintenance), which has been used extensively to plan, evaluate, and report the implementation of healthcare programs and services (38). The RE-AIM model allows for describing both implementation and dissemination processes, including the design and evaluation of specific interventions, as well as the identification of barriers and facilitators for the implementation of a health service, program, or intervention (39). The model is particularly useful to assess the implementation of health services and policies in real-world settings; it can be easily adjusted to different contexts and populations, and it has been recognized as one of the most flexible and compelling models to translate research into practice by stakeholders (http://www.re-aim.org/).

The RE-AIM model considers the following components represented by each of its letters: Reach, Effectiveness, Adoption, Implementation, and Maintenance. Reach refers to the number or proportion of potential beneficiaries that are receiving the service. Effectiveness is the impact of the different interventions offered by a patient-level program. Adoption considers the number of institutions or clinical professionals that are willing to adopt the program and use it regularly. Implementation refers to whether the program is being offered as expected or according to a particular manual, like a clinical guideline or a protocol. Finally, Maintenance is expressed at two levels: institutional (the degree to which the program is part of the regular services of a clinic) and individual (the long-term effects on people enrolled in the program) (38). To our knowledge, this is one of the few attempts to evaluate the implementation of a memory assessment service in a low- to medium-income country and the first in a South American country.

## Objective

The main objective of this study was to describe and evaluate the Reach and Effectiveness of a Memory Unit, the “Memory and Neuropsychiatry Clinic" (CMYN for its acronym in Spanish), in the Hospital Salvador (hereafter “the hospital”), a public hospital in Santiago Chile, using the RE-AIM model.

## Methodology

The methodology is divided into the following sections: Ethics, Study design, and Population including composition and internal functioning of CMYN, Data collection techniques and instruments, and Data analysis. In our study, only the “R” (Reach) and “E” (Effectiveness) components were analyzed. Other dimensions will be described in future papers.

### Ethics

In accordance with local legislation and institutional requirements, our study has been reviewed by the Research in Human Beings Ethic Committee of the Universidad de Chile's Medical School and was determinate that ethical approval was not required for this study on human participants. Neither written informed consent from the participants was required to participate in this study in accordance with the national legislation and the institutional requirements.

### Study Design and Population

To properly evaluate CMYN, some context of its history and context is needed. CMYN was established in late 2017 and began to assess patients in March 2018 in the outpatient part of the hospital, where historically the complex dementia patients had been treated, and where all the medical and non-medical specialists are based.

The Chilean Health System is divided into public and private systems, with the public system caring for over 80% of the population, especially older people. The public system has a pyramidal model of care whereby most of the specialists are hospital based and each hospital receives referrals from several areas. Hospital Del Salvador receives referral from several areas across the capital, one of which is called Peñalolen. Peñalolen is an urban area with over 240,000 inhabitants and its public health system is composed of six primary care centers, one mental health center and a daycare center for dementia. A detailed description of the Chilean health system can be found here (40).

As the Plan was in a pilot stage, it was initially decided that CMYN would only assess referrals from the primary care centers of Peñalolen, where according to the Plan, services and supports for dementia care were implemented in primary care centers as well as the provision of a Kintun, i.e., a daycare for dementia, that has been described elsewhere (8). However, since the hospital is a referral center for eight other communes in the eastern metropolitan area, CMYN quickly extended its services to patients from this larger area, regardless of whether they have facilities for dementia care within the primary care centers.

#### Study Design

Our study describes the performance of CMYN, evaluating the Reach and the Effectiveness under the RE-AIM model.

#### Population

The MU and its multidisciplinary team and specific program for dementia care received referrals from 20 primary care centers, with only six of them located in Peñalolen. CMYN receive patients mainly from primary care, referred under the diagnosis of “dementia” (confirmed) or “suspected dementia.” We also received patients from other areas of the Hospital that could be referred under any diagnosis.

### Variable/Intervention

CMYN is a multidisciplinary team of medical and non-medical specialists in dementia comprising two neurologists, two psychiatrists, a clinical psychologist, two neuropsychologists, a nurse, an occupational therapist, a speech and language therapist, and a social worker. CMYN performs several functions, including clinical evaluation and external consultation for patients with dementia and their caregivers requiring evaluation at a specialist care level, with established criteria for referral (see Table 1), management, continuity of care, and case coordination with primary care, together with research and teaching.

**Table 1:** Criteria for referral to specialist care according to the Plan.

**Criteria**	**Rapid evaluation needed**	**Diagnosis problems**	**Treatment problems**	**Communication or swallow problems**	**Caregiver burden**
	1. Convulsions 2. Rapid onset of cognitive impairment 3. History of recent falls, after emergency department evaluation	1.- Cognitive impairment including the following: 1.1.- Behavioral symptoms as an early symptom 1.2 Late onset psychiatric disorders 1.3.- Motor impairment as an early symptom 1.4.- Hallucinations and delirium as an early symptom 1.5 Cognitive fluctuations 1.6- Communication problems as an early symptom 1.7.- Neurological focal signs 2.- Rapid onset dementia (<6 months) 3.- Young onset dementia (<65 years) 4.- Subjective cognitive impairment with normal cognitive screening but impaired function	1.- Behavioral and psychological symptoms of dementia, after primary care interventions	1.- Swallow disorder 2.- Nasogastric tube complications 3.- Young onset dementia with communication disorders	1.- Caregiver with significant burden, after primary care and mental health interventions

The clinical interventions of CMYN were organized into different programs for people with dementia and their caregivers, characterized by (i) being time-limited, (ii) addressing unresolved problems after evaluation in other healthcare centers, and (iii) encouraging referral to other services in <3 months. Assessments at CMYN were organized into three programs: (1) diagnostic, (2) biopsychosocial, and (3) communication (described in Table 2), with a team leader for each program that organizes the medical and non-medical evaluation needed and being able to refer to any professional both in CMYN and the Hospital.

**Table 2:** Programs of CMYN.

**Program**	**Objective**	**Components**	**Professionals**	**Referral criteria**
Diagnostic	Making a diagnosis	Complete multidisciplinary evaluation: • Medical history • Neuropsychological assessment • Occupational therapist assessment • Blood samples • Neuroimaging • Others (as required) *Post diagnosis, there are several sessions with a clinical psychologist and social worker to help to cope with the diagnosis*	All: • Neurologist (leader) • Neuropsychologist • Clinical psychologist • Social worker Per request • Psychiatrist • Occupational therapist • Speech and language therapist	• Diagnostic evaluation • Early-onset dementia.
Biopsychosocial	Comprehensive treatment to manage symptoms related to the dementia syndrome	Multidisciplinary intervention based on the DICE (describe, investigate, create, and evaluate) model (41) • Psychopharmacological interventions • Psychological interventions for the caregiver • Occupational therapy interventions • Social worker interventions	All • Psychiatrist (leader) • Clinical psychologist • Occupational therapist • Social worker Per request • Speech and language therapist • Nurse	• Patients with severe neuropsychiatric symptoms • Caregivers with severe caregiver burden
Communication and swallowing	Evaluation and treatment of alterations in communication and/or swallowing.	Evaluation and treatment by a speech-language therapist	Speech and language therapist	• Primary Progressive aphasia • Communications and/or swallowing issues^*^

* *Most of the existing care networks do not have access to a speech therapist; the majority of patients that need a service are referred to this program*.

In exceptional circumstances, some patients were treated despite not assigned to one of the aforementioned programs, either for clinical, administrative or other reasons. These patients were kept in medical treatment, or with other professionals, according to their needs and for the time that was required. At the end of the assessment and care process, a report was created, summarizing the interventions offered, as well as making suggestions for ongoing care in the other parts of the network.

Additionally, the MU professionals train care teams from primary care centers in the diagnosis and treatment of patients with cognitive impairment and dementia. These training sessions are held every week by members of the MU and are usually delivered by a medical professional and an allied healthcare professional.

### Data Collection

Data collection has been a priority for CMYN since its inception, bearing in mind the importance of generating data that could be used to replicate and validate its implementation for use by policymakers.

To obtain accurate and more complete data, the CMYN data collection process has had to go beyond the basic clinical paper-based record system available in the hospital and has progressively developed its own electronic database. First, data were registered in an online data chart and subsequently on an electronic health registry.

The data collection was set up as follows:

1.-Registry of data: A data chart was created using Google drive, allowing it to be completed and reviewed from any device by professionals that registered data in real-time on diagnosis, treatment and referrals.

2.- The registered data were doubled checked for accuracy: First, the data of the electronic registry were compared with the paper medical record; then, our electronic registry was compared with the hospital electronic registry.

CMYN's nurse also obtained information about the dementia patients seen in Peñalolen's primary care, both referred and not referred to MU. Unfortunately, there was no reliable and systematic registry of dementia in primary care centers locate in the areas that were not part of the Dementia Plan.

To ensure patient privacy, both the electronic registry and the Google drive was protected by password, available only to CMYN's professionals. Also, for analysis, only anonymized demographic and clinical data were extracted.

### Data Analysis

The data collected from patients and clinicians were analyzed using descriptive statistics in SPSS. To compare differences between areas of referral and the discharge rates, chi-square was used.

## Results

As mentioned above, we will present only the analysis for the two first dimensions of the RE-AIM model. For Reach and Effectiveness, we provide a general evaluation regarding the performance of CMYN in each of these dimensions based on information regularly collected by healthcare professionals and interviews with key informants (e.g., patients and caregivers).

### Reach

#### Reached Patients

This dimension corresponds to the number or proportion of potential beneficiaries that are receiving the services offered by CMYN. From March 2018 up to June 2019, the *Reach* of CMYN was a total of 510 patients.

When analyzing the reached patients, most of them came from primary care (51.9%), mainly from Peñalolen (33.9% of all the patients), the pilot area of the Plan where there are specific resources for the care of people with dementia (PwD) and their families, case management, and a mandatory registry of all dementia cases. Another large group of patients came from other outpatient services of the Hospital (39.2%), particularly from the Departments of Neurology (63.3%) and Psychiatry (16.0%) of the Hospital. Eighteen percent of all the patients were referred from primary care centers of the other seven areas of the health network of the Hospital del Salvador. Only 6.2% of the patients assessed came from other secondary care outpatient services.

#### Percentual Reach

To know the percentage of patients reached by CMYN of the possible patients, some analysis is required.

In Peñalolen, 36,593 persons over 60 years are enrolled in Primary Care Centers and 120,656 in the other areas (https://degi.saludoriente.cl/degidssmo/biodemografico.php). Although dementia is not an exclusive disease of older adults, with 10% cases occurring in people under the age of 60 years, it is more common in older individuals. Considering an estimated prevalence of dementia in Chile of 7.0% in people older than 60 years old, 2,500 persons could have dementia in primary care centers in Peñalolen and 8,500 from other areas (3, 6, 42). In June 2019, 862 cases with suspected dementia (68% woman and 51% over 80 years old) were being followed up in Peñalolen's primary care center (43).

From March 2018 to June 2019, 113 cases with suspected dementia were referred from Peñalolen to CMYN, representing 5.3% of the estimated PwD of Peñalolen. The number of cases with suspected dementia in follow-up in primary care centers outside Peñalolen is not available. These centers have referred, in the same period, 304 cases of suspected dementia patients to CMYN, corresponding to <1% of PwD living in the areas of these centers.

### Effectiveness

#### National Plan of Dementia

One of the objectives of the National Plan of Dementia was to improve the diagnosis and referral rates for patients with dementia. According to the Peñalolen's primary care mental health coordinator, in the pre-Plan era, before 2018, the number of patients for follow-up with a diagnosis of suspected dementia was about 100. This number increased to over 800 from 2018 until mid-2019 (43). Considering the prevalence of dementia in Chile (3), we can estimate that at least 60% of PwD have not received a diagnosis. As mentioned before, based on our estimations, the MU has interacted with nearly 5% of PwD from the Peñalolen area.

#### CMYN Memory Unit

During the period between March 2018 and June 2019, 510 patients were referred to CMYN (143 from Peñalolen and 367 from the other areas). Ninety-one (17.8%) of the referred patients were discharged: 24 (26.3%) from the discharged came from Peñalolen and 67 (73.6%) from the other areas. There was no significant difference between the area of precedence and the rate of discharges (16.78% of patients from Peñalolen were discharged and 18.25% from those of the other areas, chi-square test: χ^2^ (1, *N* = 510) = 0.15, *p* = 0.69].

Of these 91 patients, 60 (65.93%) were referred back to primary care and 31 (34.06%) to another outpatient services at the Hospital del Salvador, mainly to Neurology and Psychiatry. Of the 91 discharged patients, 9 (10%) were also referred to a specialized mental health outpatient center, and another 9 (10%) were instructed to attend the KINTUN Daily Care Center aimed to support to people with dementia and their families.

A diagnostic of subjective cognitive complaint, or mild cognitive impairment, or cognitive complaint associated with a psychiatric disorder was made in 64 (70.4%) of the discharged patients. The diagnosis of dementia was confirmed in 24 (26.7%) that were referred to continue follow-up. Lastly, three patients (3.3%) were discharged against medical advice.

## Discussion

Our analysis on the implementation of CMYN with the Reach (number of patients) and the Effectiveness (impact on those patients) showed that the Plan reached a significant number of patients with CMYN reaching over 500 patients, with a novel and multidisciplinary approach to dementia care.

We will discuss the following key points (i) the utility of the RE-AIM to evaluate CMYN implementation; (ii) the number of patients under follow-up, (iii) comparison between CMYN and other MU worldwide, and (iv) barriers for the creations of new MU in Chile.

### The Utility of the RE-AIM to Evaluate CMYN Implementation

There are several models available to evaluate the implementation of health services and programs (44–46). Among them, RE-AIM is recommended to evaluate the implementation of health services that are still in a developmental stage and provides several other advantages to fulfill the goal of our study: it represents an easy and approachable methodology and enables us to analyze parts of the model (e.g., Reach and Effectiveness) even if the other parts are not available. The advantage of using this model is its capacity to evaluate the ongoing implementation process of CMYN and to improve and complete the evaluation as the program develops in the areas that we already analyzed as well on the other components. Our study suggests that the RE-AIM is a suitable model to evaluate the reach and effectiveness of MU and contributes to providing insight that facilitates the sharing of experiences on the setup and implementation of MU. The use of the RE-AIM model to evaluate CMYN is a unique aspect of this study. While some programs for older people have been evaluated using RE-AIM (47, 48), to the best of our knowledge, this model has not used previously to evaluate the creation of MU or the implementation of a national plan of dementia. This is probably associated to low awareness among clinicians of the need to evaluate the implementation of MU in a systematic way (44).

### The Number of Patients Being Followed Up

Our results suggest that the Plan increased the number of cases diagnosed with suspected dementia in primary care in Peñalolen from under 100 patients to over 800, suggesting that over a third of the elderly population with dementia in that area were diagnosed (43). Nevertheless, because of the lack of diagnostic confirmation of most of the cases seen at the primary care level, we were not able to calculate the exact number of PwD diagnostic in primary care, especially considering the high rate of misdiagnosis generally reported in primary healthcare (49).

Interestingly, based on our estimates, nearly 5% of all PwD of Peñalolen were referred to CMYN. That is signifiable low, especially compared with the international guidelines, like the standards from UK, that suggest that at least 66% of the dementia patients should be diagnosed by memory services (50). However, literature also suggest that referrals to MU increase significantly over the years, as MU are progressive more known among clinicians and services coordinators (50, 51). This number should be also analyzed in the context of our referral criteria, since they are different from the ones in other MU; for example, UK memory assessment services aim to evaluate all dementia patients, while our referral criteria, presented in Table 1, were atypical cases, young-onset dementia, difficult to diagnose or treat.

CMYN was able to discharge only 18% of their patients. This is not concordant with the objective of returning patents to primary care after clarification of their diagnosis and/or achieving remission of their presenting symptoms. This result can be in part explained by the relative short follow-up of our study. It is likely that more patients will be discharged over time. Nevertheless, this result suggests the existence of barriers to discharge patients from MU to primary care.

Possible barriers to discharge were that some of the medication could be prescribed and delivered only at the specialist care in MU, caregiver's resistance to discharge to primary care, and the need for longitudinal evaluations for a precise diagnosis. These barriers are similar to those reported in other studies (52, 53). One of them is the lack of specialized teams in primary care, but definitely not the only one, since most of the patients referred from primary healthcare from Peñalolen were not discharged, even having some facilities to dementia care in their primary care centers. Indeed, the rate of discharged patients from Peñalolen did not differ significantly from patients from other areas.

Future studies need to explore the low rate of discharges and possible barriers in more detail, exploring not only the discharge but also the re-entry to CMYN, since the lack of continuity of care for PwD has been associated with readmission and other poor health outcomes (54, 55), and therefore Peñalolen's patients might have fewer readmission to CMYN.

Our results also shows that even with the reported increase in reach in primary care (43), there is still a significant underdiagnosis of dementia in primary healthcare, with <50% of PwD being diagnosis according to our estimations. This is similar to the international experience (49, 56–58).

Therefore, there is a need for specific actions to improve awareness of cognitive disorders in both clinicians and the general population to increase access to opportune diagnosis and the quality of diagnostic at primary healthcare. Although frequently described in the literature (49), we can estimate the rate of misdiagnosis in primary care. On one hand, as discussed previously, the rate of referral from primary healthcare is low. On the other hand, all the referred patients from primary care came under the diagnosis of dementia to CMYN, and in over 90% of them, the diagnosis was either confirmed or is still under evaluation. Studies are needed to analyze the quality of diagnosis in primary healthcare.

One of the MU's objective is the continuous training of primary care in various aspects of dementia, including diagnosis. Our results suggest that MU need to improve its training techniques, implementing effective schemes to increase awareness and diagnosis of dementia in primary care, as has been done elsewhere (59, 60).

### Comparison Between CMYN and Other MU Worldwide

Our preliminary data are consistent with previous reports suggesting that MU are an alternative for dementia care. Although the evidence is still scarce (33), most studies show that MU deliver benefits for patients (61), caregivers (62), clinicians and health systems (63) with better outcomes than treatment as usual (25). However, as mentioned above, the data available on cost-effectiveness of MU is contradictory (26). Also, research on the implementation of MU needs to consider the existence of different models of MU that are not limited to high-income countries and big cities (29). There are reports of experiences of successful MU in low- to middle-income patients (64) and in rural areas, using telehealth evaluations (23). Other MU have been established in primary care instead of specialized care settings (65) and can be composed mainly of nurses that can assess and manage specific symptoms (66, 67). Some MU treat and manage different types of dementia such as frontotemporal dementia (68). CMYN is based in secondary care and has established a novel model of treatment for Chile, with multidisciplinary treatment sorted into several programs aimed to deal with specific issues in dementia care such as diagnosis and treatment, with a continuous support for primary care. Furher research is needed to study if this new model generates better outcomes than treatment as has been done before in Chile and is more cost-effective.

### Barriers for the Creations of New MU in Chile

We have identified several barriers, mainly related to the creation of multidisciplinary teams, coordination of care, funding, and health policy, to the creation of MU in Chile.

In Chile, there is no history and tradition of multidisciplinary teams operating in specialized care settings. Traditionally, PwD have been treated only by the neurologist or geriatrician using a biomedical and mainly pharmacological approach, with little or no interaction with other medical or non-medical health professionals. The implementation of a multidisciplinary team to tackle dementia might be seen as unnecessary.

At a coordination level, the lack of facilities for care of PwD at primary care centers, well-defined primary and specialist care referral pathways and processes, and a shared EHR, as mentioned above, are barriers to the implementation of MU due to problems with developing effective referral and discharge processes for the PwD from MU to primary care centers patients (69). Also, training and education in primary care are critical to improve referral and discharge and assure continuity of care in primary healthcare (70). Regarding prescription delivery, the creation of a joined-up service could facilitate the medication continuity in the transition from specialist care to primary based. That is one of the changes, among others, needed toward building capacity for dementia care in primary care, as it has been suggested elsewhere (71, 72).

At a funding level, a fee-for-service (FFS) payment model in healthcare is a barrier to care based on multidisciplinary teams. Supervision, management and discussion of cases, and coordination of care are not reimbursed in FFS (73). Indeed, current evidence suggests that value-based payment models are more suitable for MU (19).

At the health policy level, the main barrier is the lack of continuity of health policy regarding dementia in Chile. The plans for dementia care changes along the different governments as well as the priority of dementia among other health conditions. Hence, the funding for the creation of MU, daycare centers, and training program is not guaranteed to remain stable over the years.

## Limitations

The main limitation of our study was the lack of information before and after the MU interventions in most of the areas. Our results suggest that the implementation of Plan increased the number of cases with suspected dementia diagnosed by GPs at primary care in Peñalolen. Nevertheless, we did not have more reliable information on the care of PwD before the implementation of MU to evaluate change in the navigation pathway at the whole health network associated with the implementation of the Dementia Plan and MU. The lack of availability of this information is mainly explained by the configuration of the Chilean health system: primary care is separated from secondary care with respect to data registry, clinical guidelines, and administration.

Another limitation is the availability of data. There is no shared EHR either between areas of the hospital or primary care. It is very difficult with the current registries to map the journey of a PwD across health systems. We need to create a shared dementia registry using EHR that would enable better tracking, evaluation, and care for PwD, as has been described elsewhere (74).

We were not able to evaluate the AIM (adoption, implementation, maintenance) components of the RE-AIM model, mainly due to the early phase of the implementation of the National Plan of Dementia and CMYN.

To evaluate adoption, other centers need to replicate the interventions made in our MU. Although there have been some informal discussions regarding adoption of our MU model in other hospitals, this has not occurred yet.

To evaluate implementation in the RE-AIM model, as described above, a clinical guideline is required prior to the start of the program to evaluate if the program follows it. This is not possible, due to the fact that the Chilean Ministry of Health has neither specified the organization of MU nor provided a manual or protocol for the creation of MU. Hence, a challenge, for CMYN, is to design a manual for the implementation of MU based on our experience.

Finally, to evaluate maintenance over the years, more time is required and due to the short time CMYN has been operating, we are not yet able to evaluate this phase.

## Conclusion

A collaborative care model for dementia has been recommended, integrating primary care, specialist care, and national policies on dementia (20, 75, 76) and has been proven to be cost-effective (77). MU have been proposed as a significant part of the dementia care strategies. Due to the increasing prevalence of dementia, WHO currently recommends that the majority of dementia patients should be treated in primary care in coordination with secondary care and only the more complex patients should be referred to MU (78). MU clinics are recommended even in low-income countries (28, 79). The implementation of the Plan was a significant step forward for dementia care in Chile (40). However, in the Plan, there are no recommendations or guidelines on how to organize MU and how to interact with primary care.

As a result, even if the three MU in Chile have the same objective, they present significant differences in terms of human resources, health programs, and links with primary and secondary care. Even though it is important for the MU to adapt to local reality, there is also the need for harmonized practices between teams. With a common practice, it is easier to improve quality of care, gather evidence for further implementation of MU, and develop research, as has been recommended elsewhere (35, 69, 80).

To our knowledge, there are no guidelines in Latin America on how to implement MU. Although cultural differences and differences in healthcare organization worldwide might play a role in the lack of international guidelines, the publication of the evaluation of the first year of CMYN generates some useful information and creates a model of intervention that could be helpful not only for the expansion of MU across Chile but also for any Latin American country looking to implement a national dementia plan.

Further studies are needed to assess the contribution of MU in comparison to regular care in the Chilean health system, as it has been proven elsewhere (81) and to develop a model of care that assures continuity and coordination of care in the health sector, avoiding discontinuation of care when the case is transferred from primary to secondary care and back.

Also, it is important to keep a constant process of evaluation and improvement in all health services to both evaluate the efficacy and sustainability of the intervention and use that information to generate better interventions. The lack of health registry hampers the evaluation of a health program (82), since it makes it harder to gather standardized data for analysis.

Finally, in dementia care, there is a need for a constant process of adaptation due to the heterogeneity of patients (83–85), their caregivers (10), and their needs (86). However, there is also a heterogeneity for clinical and administrative reasons, the quality and quantity of resources, the assigned population to each hospital (as happened to CMYN), and the different professionals' background and experience.

Therefore, a clinical protocol should always be fluid and adaptable, looking to create a tailor-made intervention for each reality. Research should focus on enhancing the knowledge on how to implement MU, aiming to create profiles of cases and guidelines to respond using evidence-based interventions that are both effective and sustainable.

## Data Availability Statement

The data analyzed in this study is subject to the following licenses/restrictions: Electronic record of clinical interventions. Requests to access these datasets should be directed to tileon@uc.cl.

## Ethics Statement

Ethical review and approval was not required for the study on human participants in accordance with the local legislation and institutional requirements. Written informed consent from the participants was not required to participate in this study in accordance with the national legislation and the institutional requirements.

## Author Contributions

TL and AS: conception. TL, AS, LC, and FM: design. LC and TL: analysis. TL, AS, BL, and FM: interpretation. TL, LC, FM, BL, and AS: draft manuscript and substantial manuscript revision. All authors contributed to the article and approved the submitted version.

## Author Disclaimer

The contents of this publication are solely the responsibility of the authors and do not represent the official views of the sponsors.

## Conflict of Interest

The authors declare that the research was conducted in the absence of any commercial or financial relationships that could be construed as a potential conflict of interest. The reviewer MH declared a shared affiliation, with no collaboration, with one of the authors AS to the handling Editor at time of review.

## Publisher's Note

All claims expressed in this article are solely those of the authors and do not necessarily represent those of their affiliated organizations, or those of the publisher, the editors and the reviewers. Any product that may be evaluated in this article, or claim that may be made by its manufacturer, is not guaranteed or endorsed by the publisher.
